# Anti-Inflammatory Effects of Immunostimulation in Patients with COVID-19 Pneumonia

**DOI:** 10.3390/jcm10245765

**Published:** 2021-12-09

**Authors:** Pierachille Santus, Dejan Radovanovic, Micaela Garziano, Stefano Pini, Giuseppe Croce, Giuseppe Fuccia, Debora Spitaleri, Mara Biasin, Mario Clerici, Daria Trabattoni

**Affiliations:** 1 Department of Biomedical and Clinical Sciences (DIBIC), Università degli Studi di Milano, Via G.B. Grassi 74, 20157 Milan, Italy; pierachille.santus@unimi.it (P.S.); micaela.garziano@unimi.it (M.G.); stefano.pini@unimi.it (S.P.); giuseppe.croce@unimi.it (G.C.); giuseppe.fuccia@unimi.it (G.F.); debora.spitaleri@unimi.it (D.S.); mara.biasin@unimi.it (M.B.); 2 Division of Respiratory Diseases, Luigi Sacco University Hospital, ASST Fatebenefratelli-Sacco, Via G.B. Grassi 74, 20157 Milan, Italy; dejan.radovanovic@asst-fbf-sacco.it; 3 Department of Pathophysiology and Transplantation, Università degli Studi di Milano, Via Francesco Sforza 35, 20122 Milan, Italy; mario.clerici@unimi.it; 4 Don Carlo Gnocchi Foundation, IRCCS, 20148 Milan, Italy

**Keywords:** COVID-19, viral pneumonia, immune response, pidotimod, immunomodulation, respiratory failure, toll like receptor, cytokine

## Abstract

Background: The effects of immunomodulators in patients with Coronavirus Disease 2019 (COVID-19) pneumonia are still unknown. We investigated the cellular inflammatory and molecular changes in response to standard-of-care + pidotimod (PDT) and explored the possible association with blood biomarkers of disease severity. Methods: Clinical characteristics and outcomes, neutrophil-to-lymphocyte ratio (NLR), plasma and cell supernatant chemokines, and gene expression patterns after SARS-CoV-2 and influenza (FLU) virus in vitro stimulation were assessed in 16 patients with mild-moderate COVID-19 pneumonia, treated with standard of care and PDT 800 mg twice daily (PDT group), and measured at admission, 7 (T1), and 12 (T2) days after therapy initiation. Clinical outcomes and NLR were compared with age-matched historical controls not exposed to PDT. Results: Hospital stay, in-hospital mortality, and intubation rate did not differ between groups. At T1, NLR was 2.9 (1.7–4.6) in the PDT group and 5.5 (3.4–7.1) in controls (*p* = 0.037). In the PDT group, eotaxin and IL-4 plasma concentrations progressively increased (*p* < 0.05). Upon SARS-CoV-2 and FLU-specific stimulation, IFN-γ was upregulated (*p* < 0.05), while at genetic transcription level, Pathogen Recognition Receptors (TRLs) were upregulated, especially in FLU-stimulated conditions. Conclusions: Immunomodulation exerted by PDT and systemic corticosteroids may foster a restoration in the innate response to the viral infection. These results should be confirmed in larger RCTs.

## 1. Introduction

Severe acute respiratory syndrome coronavirus type 2 (SARS-CoV-2) is the pathogen that causes the Coronavirus Disease 2019 (COVID-19), a pandemic pathology currently affecting almost 200 million patients worldwide. The clinical manifestations of COVID-19 are very heterogeneous, ranging from mild forms with uncomplicated illness to critical cases associated with significant in-hospital mortality, characterized by severe bilateral pneumonia leading to acute respiratory distress syndrome (ARDS) and the need for mechanical ventilation [1,2,3,4].

Studies on the pathogenesis of the host-viral response have shown that the SARS-CoV-2 infection leads to a local immune response, recruiting macrophages and monocytes that respond to the infection, releasing cytokines and prime adaptive T and B cell immune responses [5]. The observation that pro-inflammatory cytokines and chemokines, including Interleukin-6 (IL-6), Interferon-γ (IFNγ), Monocyte Chemoattractant Protein-1 (MCP-1), and Interferon gamma-induced protein 10 (IP-10), are massively produced in severe COVID-19 indicate the presence of a T helper 1 (TH1) cell-polarized response, resulting in the homing of monocytes and T lymphocytes, but not neutrophils, into infected sites [5]. In these patients, absolute counts and percentages of lymphocytes including CD4 Lymphocytes T (CD4+ T), CD8 cytotoxic lymphocytes (CD8+ cytotoxic T), natural killer (NK), and B cells are reduced as well, possibly as a consequence of both the direct cell death secondary to viral infection, and the exhaustion and depletion of T cells driven by circulating chemokines. B cells are also central in the immune response against SARS-CoV-2 infection, and some lines of evidence have hypothesized that the role of antibody-dependent enhancement (ADE) may interfere with neutralizing the antibody response [6]. Finally, the function of both monocytes and macrophages is altered in COVID-19 patients, in whom neutrophilia due to increased numbers of mature and immature cells is observed as well. 

In vivo and in vitro studies show that Pidotimod’s (PDT) immunomodulatory activity targets both adaptive and innate immunity. PDT induces dendritic cells (DCs) maturation, up-regulates the expression of Human Leukocyte Antigen—DR isotype (HLA-DR) and co-stimulatory molecules, stimulates DCs to release pro-inflammatory molecules that drive T-cells proliferation and differentiation towards a Th1 phenotype, enhances NK cells functions, and promotes phagocytosis [7,8]. Supplementation of antibiotic therapy with PDT in both adult and pediatric patients affected by community acquired pneumonia resulted in an upregulation of antimicrobial and of immunomodulatory proteins as well as in an increased percentage of Toll-Like Receptor type 2 (TLR2)—and TLR4, as well as CD80- and CD86-expressing immune cells [9,10]. Recent results also showed PDT could rebalance pro- and anti-inflammatory cytokines and induce a significant reduction of cystatin C levels in HIV-infected patients [11]. Moreover, in an outpatient population affected by SARS-CoV2 infection, PDT reduced the duration of symptoms with an earlier defervescence [12], suggesting that the complex effects of PDT on the immune response could be beneficial in SARS-CoV-2 infection. 

We investigated in patients with COVID-19 pneumonia and mild to moderate respiratory failure whether the immunomodulatory activity of PDT could beneficially modulate immune responses and if this could be associated with changes in serum inflammatory biomarkers and improved clinical outcomes.

## 2. Materials and Methods

### 2.1. Study Design

This was a prospective, observational, exploratory, matched historical cohort study conducted in the High Dependency Respiratory Unit of Luigi Sacco University Hospital, a secondary care teaching hospital in Milano, Italy. Adult patients hospitalized with microbiologically and radiologically confirmed COVID-19 pneumonia were consecutively enrolled between October 2020 and January 2021. Patients anthropometrical and clinical characteristics were collected from digital records. Vital signs and blood gas analyses were collected on daily basis. 

Serum inflammatory biomarkers and biochemistry were collected at patients’ arrival in the emergency department, at admission in the HDRU, and every 2 to 3 days depending on patients’ clinical course until discharge from the unit. In patients exposed to PDT, blood samples for cytokine and transcriptomic analyses were drawn the day PDT was started before first PDT administration, at 7 +/− 1 days (T1), and at 12 +/− 1 days (T2) of treatment. Major and minor adverse events were recorded on a daily basis.

The study protocol (ClinicalTrials.gov: NCT04307459), designed following the amended declaration of Helsinki (2013), was approved by the local ethical committee (Comitato Etico Milano Area I; 17263/2020) and all recruited patients gave written informed consent.

### 2.2. Patients’ Characteristics 

The diagnosis of COVID-19 pneumonia was based on a naso-pharyngeal swab that tested positive for SARS-CoV-2 (reverse transcriptase polymerase chain reaction—RT-PCR) and on the presence of pulmonary infiltrates at the chest X-ray or CT scan performed in the emergency department [13].

Patients intubated in the first 24 h after admission in the HDRU and with signs of severe acute respiratory distress syndrome (arterial partial pressure of oxygen to inspired oxygen fraction ratio (PaO_2_/FiO_2_) < 150 mmHg while receiving 5 cmH_2_O of positive end expiratory pressure [14]) were excluded. Patients were also excluded if: (1) they were receiving immunosuppressive therapy at the time of hospital admission (e.g., long term systemic corticosteroids, methotrexate, mycophenolate mofetil); (2) they were undergoing or received in the last 5 years radio, chemo, or immune therapy for solid or hematologic malignancies; (3) patients with history of rheumatologic diseases or acquired/genetic immune disorders; (4) women in childbearing age; (5) they had a history of drug or alcohol abuse; (6) they were receiving direct anticoagulants, warfarin or acenocoumarol at the time of hospital admission; (7) they received off-label treatment with remdesivir or tocilizumab during the hospital stay; (8) they had a mental illness or dementia that prevented them from understanding the study procedures and signing the informed consent. 

### 2.3. In-Hospital Treatment 

Patients that satisfied criteria for severe pneumonia according to the American Thoracic Society/Infectious Diseases Society of America (ATS/IDSA) guidelines [15] received in vein methylprednisolone at a maximal dose of 1 mg/kg as also suggested by Salton and coworkers [16] and as previously published [1,13]. According to national recommendations [17], and as reported elsewhere [1,13], unless contraindicated, all patients received prophylactic low molecular weight heparin (LMWH) at admission. If signs of deep vein thrombosis or pulmonary embolism were detected or patients showed D-dimer values > 3000 mg/L fibrinogen equivalent units (FEU), a therapeutic dose of LMWH was introduced. Antibiotic therapy was administered in case of suspected super-infection or a bacterial pathogen was isolated during the hospital stay. 

Patients with a PaO_2_/FiO_2_ < 300 mmHg while receiving oxygen with a FiO_2_ > 50% by means of Venturi or reservoir masks and that showed signs of respiratory distress, a respiratory rate > 30/min, received continuous positive expiratory pressure delivered by high-flow generators using a helmet as an interface as previously reported [1,13,18,19]. Criteria for instituting weaning from continuous positive airway pressure (CPAP), CPAP failure, and eligibility for invasive mechanical ventilation were reported elsewhere [18,20].

### 2.4. Pidotimod and Historical Control Group

Since October 2020 (beginning of study enrollment), the standard operating procedures of the HDRU included PDT 800 mg twice daily as part of the treatment of patients with COVID-19 pneumonia. Patients that received PDT during the hospital-stay were matched with a historical cohort of patients with COVID-19 pneumonia hospitalized in our HDRU between March and October 2020 that did not receive PDT as part of the in-hospital bundle of care. Patients included in the control group were matched according to anthropometrical characteristics, severity of pneumonia, and symptoms onset. Specifically, criteria for matching were: age (+/−2 years), gender, PaO_2_/FiO_2_ at admission (+/−10%), C-reactive protein at admission (+/−20%), and days since symptoms onset at admission (+/−2 days).

### 2.5. PBMC Isolation and Stimulation

Whole blood was collected from patients in Ethylene Diamine Tetra Acetic acid (EDTA) tubes (BD Vacutainer, San Diego, CA, USA) at three different time points (T0 = before first PDT administration; T1 = after 7 +/− 1 days of PDT treatment; and T2 = 12 +/− 1 days of PDT treatment). Samples were centrifugated at 1200 rpm for 10 min; plasma obtained was collected and stored at −20 °C for subsequent analysis. Peripheral blood mononuclear cells (PBMCs) were isolated by density gradient centrifugation on Ficoll (Cedarlane Laboratories Limited, Hornby, ON, Canada), and viable cells were counted with the automated cell counter ADAM-MC (Digital Bio, NanoEnTek Inc., Seoul, KR, Korea). PBMCs were resuspended at the concentration of 1 × 10^6^/mL in RPMI 1640 medium (Euroclone, Milan, Italy) supplemented with 10% fetal bovine serum, 1% of L-glutamine (LG), and 2% pen-streptomycin (PS). 

SARS-CoV-2 viral stock were inactivated at 60 °C for 30 min and 1 MOI of the virus was used to stimulate 1 × 10^6^ PBMCs. An Influenza virus (FLU) vaccine prepared with a mixture of an inactivated trivalent subunit formulation that contains the hemagglutinin antigens of influenza A H1N1, influenza A H3N1, and influenza B virus strains (each at 30 mg/mL; final dilution, 1/1000) was used to evaluate recall immune responses in vitro. For gene expression, cells were harvested 10 h after stimulation while cytokine content was quantified on cell culture supernatants after 24 h of stimulation.

### 2.6. Multiplex Cytokine Analyses

A 27-cytokine multiplex assay was performed on plasma and cell culture supernatants, 24 h after PBMCs stimulation with inactivated SARS-CoV-2 (iSARS-CoV-2), as described above, using magnetic bead immunoassays (Bio-Rad, Hercules, CA, USA) and Luminex 100 technology (Luminex, Dallas, TX, USA) according to the manufacturer’s protocol. 

### 2.7. Quantigene Plex Gene Expression Assay

Gene expression of 10 × 10^5^ PBMCs was performed by quantiGene Plex assay (Thermo Scientific, Waltham, MA, USA), which provides a fast and high-throughput solution for multiplexed gene expression quantitation, allowing for the simultaneous measurement of 69 custom selected genes of interest in a single well of a 96-well plate. The QuantiGene Plex assay is hybridization-based and incorporates branched DNA (bDNA) technology, which uses signal amplification for direct measurement of RNA transcripts. The assay does not require RNA purification.

### 2.8. Neutrophil to Lymphocyte Ratio

Neutrophil to lymphocyte ratio (NLR) was reported as a prognostic marker of disease severity and mortality in COVID-19 patients [21,22,23]. Different cut off values have been proposed to categorize patients at higher or lower risk of unfavorable outcomes. A recent meta-analysis [24] showed that an NLR value of ≥4.5 had a sensitivity of 0.74 and a specificity of 0.86 for predicting disease severity, and was applied to the present study.

### 2.9. Study Outcomes 

The primary outcome of the study was to investigate the cellular inflammatory and molecular changes in response to PDT during the hospital stay and to explore the possible association with blood biomarkers of disease severity such as NLR and D-dimer values. Secondary outcomes were to analyze the clinical evolution of patients that received PDT and compare it with the historical matched cohort in terms of severity of respiratory failure and need for CPAP after 7 days of hospitalization, then length of hospital stay. 

### 2.10. Statistical Analysis 

Based on the exploratory nature of the study and considering the absence of relevant or similar studies in literature, the study power could not be assessed and the sample size calculation was not possible. Considering the strict inclusion and exclusion criteria and given the dynamics of the pandemic in Northern Italy, we planned to include at least fifteen patients in the intention to treat analysis.

Qualitative variables were summarized with absolute and relative (percentage) frequencies. Parametric and non-parametric quantitative variables were described with means (standard deviation—SD) and medians (Inter Quartile Range—IQR), respectively. Fisher’s exact and χ^2^ tests were used to compare qualitative variables, whereas Student’s *t*-test or Mann–Whitney U test, and the analysis of variance or Kruskal–Wallis, corrected with Sidak adjustment, were used to compare quantitative variables with normal or non-normal distribution, respectively. Cox proportional hazard regression analysis was performed to assess the relationship between clinical outcomes and independent variables. A two-tailed *p* value < 0.05 was considered statistically significant. All statistical computations were performed with IBM SPSS Statistics for Windows, version 23 (IBM Corp., Armonk, NY, USA).

For immunological analyses, the Student’s t test, the χ^2^ method, and Fisher’s exact test were applied when appropriate for statistical analysis to compare continuous and categorical variables. A *p*-value < 0.05 was chosen as cutoff for significance. Data were analyzed with GraphPad Prism version 8 (La Jolla, CA, USA).

## 3. Results

### 3.1. Patients’ Clinical Characteristics

Sixteen patients (8M, 8F; median age 60 years) were enrolled in the study. Eight (50%) patients had arterial hypertension and 4 (25%) had diabetes mellitus (Table 1). At admission, all patients had respiratory failure, which was mild in 7 (44%) and moderate in 9 (56%) cases. During hospitalization, 4 patients were treated with CPAP. PDT was administered 1 (1–1.5) days after hospital admission, corresponding to 7 (5–11.5) days from symptom onset (Table 1).

Compared with patients that received PDT, historical controls were not significantly different in terms of anthropometrical and clinical characteristics, comorbidities, and severity of pneumonia at admission.

At 7 days post-admission, 11 (69%) patients had a PaO_2_/FiO_2_ > 300 mmHg, while 5 (31%) had still a moderate respiratory failure. Ten patients were weaned from oxygen therapy and 3 were successfully weaned from CPAP.

### 3.2. Clinical Outcomes

The median length of hospital stay was 10 (8–14) days and was not different as compared with the control group. None of the patients in the PDT group underwent invasive mechanical ventilation or died during the hospitalization, while one patient in the control group was intubated, transferred in the ICU, and eventually died. No major or minor adverse event was registered in patients treated with PDT.

### 3.3. Neutrophil to Lymphocyte Ratio

At admission, patients treated with PDT had a median (IQR) NLR of 7.45 (2.7–12.9), and 10 (62%) had a NLR > 6.5, not significantly different as compared to the historical control group (Table 2). Since the initiation of PDT, the NLR tended to decrease and was significantly different after 7 days of treatment in the PDT group (median (IQR) NLR 6.35 (2.3–9.3) vs. 2.9 (1.7–4.6); *p* < 0.001), with the latter being significantly different compared with the NLR observed in the control group (2.9 (1.7–4.6) vs. 5.5 (3.4–7.1); *p* = 0.037) (Table 2). Accordingly, the proportion of patients with a NLR ≥ 6.5 was reduced from 8 (50%) to 1 (6%) at 7 days post PDT initiation, significantly less compared to the control group (6 patients—37%, *p* = 0.033) (Table 2).

### 3.4. Cytokine and Chemokine Plasma Levels

**Figure 1:**
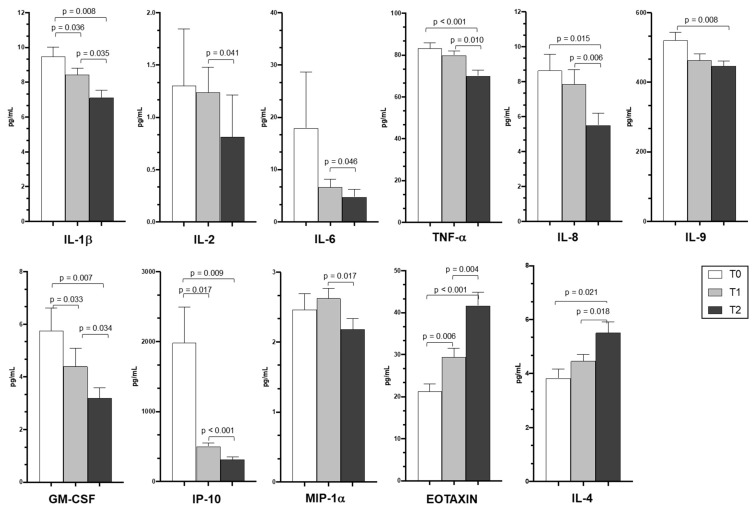
Circulating cytokine and chemokine profile of PDT-treated patients. Plasma cytokine/chemokine concentrations (pg/mL) in PDT-treated patients analyzed at baseline (white bars), at T1 (light grey bars), and at T2 (dark grey bars). Mean and standard error values and statistically significant differences (*p* < 0.05) are indicated. T0 = at admission; T1 = after 7 days of PDT treatment; T2 = after 15 days of PDT treatment. IL: interleukin; TNF-α: tumor necrosis factor-α; GM-CSF: granulocyte-macrophage colony-stimulating factor; IP-10: Interferon-gamma inducible Protein-10; MIP-1α: macrophage inflammatory protein 1 alpha.

Cytokine and chemokine concentrations were measured in plasma samples collected from patients with COVID-19 pneumonia exposed to PDT. Plasma concentration of inflammatory cytokines and chemokines, including IL-1β, IL2, IL-6, tumor necrosis factor-α (TNF-α), granulocyte-macrophage colony-stimulating factor (GM-CSF), Interferon-gamma inducible Protein-10 (IP-10), and macrophage inflammatory protein 1α (MIP-1α), were generally significantly reduced at T1 and T2 (*p* < 0.05) compared to T0 (Figure 1). Eotaxin and IL-4 plasma concentration, on the other hand, progressively increased during the study period. 

### 3.5. SARS-CoV-2 Specific Immune Profile 

PBMCs of all subjects enrolled in the study were incubated in the presence/absence of SARS-CoV-2 antigens to analyze cytokine production and gene expression; stimulation with FLU vaccine was used as a positive control. 

**Figure 2:**
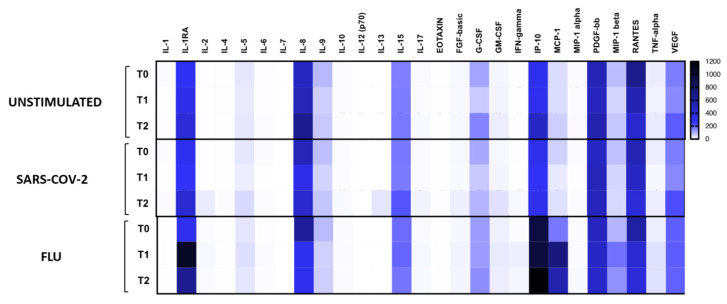
Virus-specific cytokine and chemokine production. Cytokine and chemokine production was analyzed in culture supernatants of PBMCs from Pidotimod-treated patients at baseline, T1, and at T2 in unstimulated condition or upon stimulation with SARS-CoV-2 or FLU vaccine. Heatmaps of mean values are indicated.

TNF-α, MIP-1α, MIP-1β, and IL-5 production was significantly reduced during PDT treatment (*p* < 0.05) (Figure 2 and Table 3) both upon SARS-CoV-2- and FLU-specific stimulation, while production of IFN-gamma was upregulated (*p* < 0.05) (Figure 2 and Table 3). This result was further confirmed by the gene expression data.

**Figure 3:**
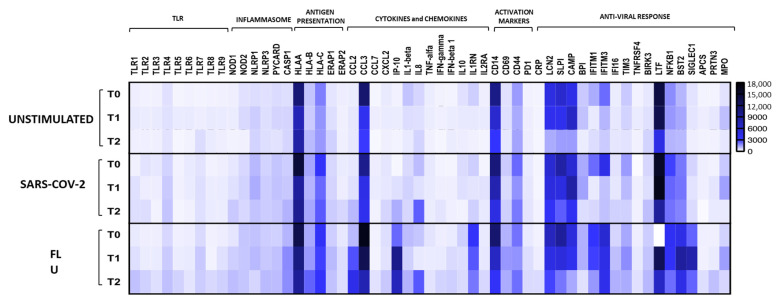
Immune-genes signature of PBMCs. Immune genes status was analyzed in PBMCs from PDT-treated patients at baseline, T1, and at T2 in unstimulated condition or upon stimulation with iSARS-CoV-2 or FLU vaccine. Heatmaps of mean values are indicated.

At the gene expression level, PBMCs from PDT-treated patients displayed an up-regulation of Toll-Like Receptors (TLR1, TLR2, TLR3, TLR4, TLR5, TLR8, and TLR9), which reached statistical significance in the FLU-stimulated condition (Figure 3).

In keeping with cytokine quantification data, gene expression results confirmed that circulating lymphocytes from PDT-treated patients were characterized by a marked decrease of pro-inflammatory cytokines (IL-1β, MIP-1alpha, and TNF-alpha) (Figure 3 and Table 3). Moreover, CD14-gene expression was strongly reduced during PDT in all conditions of stimulation.

## 4. Discussion

The main findings of the present study can be summarized as follows: (1) in patients treated with PDT, a wide array of inflammatory cytokines/chemokines significantly decreased both at circulating level and in response to specific SARS-CoV-2 and FLU stimulation; (2) eotaxin plasma levels and production of IFN-gamma in FLU stimulated PBMCs progressively increased during the study period; (3) compared with the matched historical control group, patients treated with PDT showed a significant reduction in the NLR; (4) no significant difference in clinical outcomes was observed in patients treated with PDT compared with historically matched controls.

To our knowledge, this is the first study that investigated the employment of immunostimulation during the acute phase of COVID-19 pneumonia. Indeed, Ucciferri and colleagues [12] have demonstrated that the administration of PDT in patients with mild SARS-CoV-2 infection without signs of pneumonia or respiratory failure was able to reduce the time to clinical resolution compared with untreated controls. In our study, all patients had mild to moderate respiratory failure and the standard of care included PDT on top of systemic corticosteroids and low molecular dose heparin. As expected, we observed a progressive reduction of cytokines such as IL-1, IL-2, IL-6, TNF-α, IL-8, IL-9, GM-CSF, IP-10, and MIP-1α, which was most probably secondary to the down-regulation of the inflammatory response promoted by systemic corticosteroids. Marked up regulation of IL-2, MIP1-α, and IP10 have been related to fatal COVID-19 pneumonia [25]. Interestingly however, we also observed a concomitant significant increase in eotaxin and IL-4 plasma levels. Eotaxin is an eosinophil-specific chemokine associated with the recruitment of eosinophils into sites of inflammation [26], and increased eotaxin concentration was recently associated with favorable clinical outcomes in patients hospitalized with COVID-19 pneumonia [27]. Accordingly, IL-4 plays an important role in TH2 driven inflammatory responses [28] and has been implicated in the altered inflammatory response to SARS-CoV-2 infection and lung remodeling [29].

Patients exposed to PDT also showed an increased production in IF N-γ, especially in response to FLU stimulation. An impaired IFN-gamma production due to the interference of SARS-CoV-2 with the mechanisms regulating innate immunity has been identified as one of the key factors in the altered immune response to the viral infection [30]. PDT has been previously shown to potentiate the innate immune response and antimicrobial activity both in animal models and in children with Down syndrome after influenza vaccination [31,32]. Moreover, it is known to express positive immunomodulatory effects in children and adults hospitalized with community acquired pneumonia [9,10], exerting an up regulation of TLR2 and TLR4 [9]. In line with these data, we observed that PDT-treated patients displayed an up-regulation of TLR1, TLR2, TLR3, TLR4, TLR5, TLR8, and TLR9, especially in FLU-stimulated conditions. 

Clinical outcomes such as length of hospital stay, in-hospital death, and need for invasive mechanical ventilation or admission to ICU in patients that received PDT did not differ compared with the historically matched patients. However, at 7 days post admission, PDT-treated patients experienced a more rapid recovery of respiratory failure, with a reduction of the NLR and a significant increase in lymphocyte counts. Several recent studies have demonstrated that the NLR can be used as a predictor of disease severity, progression, with high NLR being associated with higher mortality risk [24,33]. A recent systematic review including 2967 patients with COVID-19 pneumonia showed that NLR had a pooled sensitivity and specificity to predict mortality of 0.83 and 0.83, respectively [24]. Although the historical comparison limits the speculation on these results, we hypothesize that a more rapid restoration of circulating lymphocytes (lower NLR), and thus a reduced portion of lymphocytes homing into the pulmonary district, could be explained by the reduced expression of pro-inflammatory cytokines and a decrease of pro-inflammatory phenotypes of circulating lymphocytes (IL-1β, MIP-1γ, and TNF-γ) that we observed in patients exposed to PDT.

### Study Limitations

The present study has several limitations. First, the lack of a sham control group limits the distinction between the immunomodulatory effects of PDT and the effects of systemic corticosteroids in the acute phase of a COVID-19 pneumonia. However, the present observations reflect the standard of care active at the time of data collection and could be only matched with a convenience historical cohort of patients treated with systemic corticosteroids alone, thus limiting the external generalizability of the results. Second, the sample size was limited, and may have impaired the chance to observe definite correlations between immunological data and clinical outcomes.

## 5. Conclusions

This is the first study that investigated the immune profile of patients with mild/moderate COVID-19 pneumonia exposed to PDT. Our data suggest that the immunomodulation exerted by PDT in addition to systemic corticosteroids may foster a restoration in the innate response to the viral infection, exerted by the up-regulation of TLRs and the restoration of the IFN-γ response. These preliminary results may suggest a favorable role of immunomodulation in patients with SARS-CoV-2 infection, and should be confirmed in larger randomized controlled trials.

## Figures and Tables

**Table 1:** Clinical characteristics and hospitalization outcomes of the Pidotimod and control groups.

	Pidotimod (*N* = 16)	Controls (*N* = 16)	*p*-Value
Males, *n* (%)	8 (50)	8 (50)	1.000
Age, years	60 (55–71)	61 (54–69)	0.867
Arterial hypertension, *n* (%)	9 (56)	8 (50)	0.719
Diabetes mellitus, *n* (%)	4 (25)	4 (25)	1.000
Ischaemic heart disease, *n* (%)	3 (19)	3 (19)	1.000
COPD, *n* (%)	2 (13)	1 (6)	0.310
From symptoms onset to admission, days	6 (4–10)	8 (5–11)	0.323
**Variables at admission**			
PaO_2_, mmHg	73 (66–90)	90 (83–140)	0.005
PaO_2_/FiO_2_, mmHg	218 (165–289)	244 (168–294)	0.669
PaO_2_/FiO_2_ 200–300 mmHg, *n* (%)	7 (44)	6 (37)	0.719
PaO_2_/FiO_2_ 100–200 mmHg, *n* (%)	9 (56)	10 (63)	0.719
CPAP, *n* (%)	4 (40)	6 (37)	0.446
Glasgow coma scale, score	15 (15–15)	15 (15–15)	0.780
C reactive protein, mg/L	50 (24–138)	69 (41–148)	0.381
D-dimer, mg/L FEU	558 (421–811)	787 (574–1362)	0.070
From admission to PDT start, days	1 (1–1.5)	--	--
From symptoms to PDT start, days	7 (5–11.5)	--	--
**Variables 7 days post admission**			
PaO_2_, mmHg	74 (66–87)	72 (67–83)	0.953
PaO_2_/FiO_2_, mmHg	342 (288–380)	273 (196–338)	0.033
PaO_2_/FiO_2_ 200–300 mmHg, *n* (%)	0	4 (25)	0.033
PaO_2_/FiO_2_ <200 mmHg, *n* (%)	5 (31)	6 (37)	0.710
C reactive protein, mg/L	50 (24–138)	69 (41–148)	0.381
D-dimer, mg/L FEU	661 (409–951)	764 (585–1183)	0.196
**In-hospital treatments**			
Systemic corticosteroids	15 (94)	12 (75)	0.144
Antibiotics, *n* (%)	3 (19)	8 (50)	0.063
LMWH, *n* (%)	16 (100)	16 (100)	0.310
Prophylactic dose, *n* (%)	11 (69) *	14 (87) *	0.200
Therapeutic dose, *n* (%)	6 (37)	4 (25)	0.446
**Clinical outcomes**			
CPAP at 7 days, *n* (%)	1 (6)	4 (25)	0.144
Invasive mechanical ventilation, *n* (%)	0	1 (6)	0.310
Tranferred to ICU, *n* (%)	0	1 (6)	0.310
Lenght of stay, days	10 (8–14)	11 (8–21)	0.770
From symptoms to discharge, days	17 (13–23)	18 (16–31)	0.358
Death HDRU, *n* (%)	0	0	--
Death ICU, *n* (%)	0	1 (6)	0.310
Discharged to low intensity, *n* (%)	1 (12)	7 (44)	0.014

Data are reported as frequencies (prevalence) and medians (inter-quartile ranges) as appropriate. * Two patients in the control group and one patient in the Pidotimod group were shifted from prophylactic to therapeutic doses of low molecular weight heparin (LMWH) during the hospitalization.

**Table 2:** White blood cell counts and lymphocytes distribution at admission and after 7 days of hospitalization in patients that received Pidotimod and in controls.

	Pidotimod (*N* = 16)	Controls (*N* = 16)	*p*-Value
**At admission**			
WBC count at admission, ×10^6^/µL	7450 (5990–10,840)	6010 (5380–11,740)	0.520
Neutrophil count, ×10^6^/µL	8580 (5150–11,010)	8740 (4800–11,890)	0.670
Lymphocyte count, ×10^6^/µL	750 (590–1350)	820 (610–1610)	0.520
NLR	7.45 (2.7–12.9)	6.85 (4.1–10.3)	0.809
NLR ≥ 6.5	10 (62)	8 (50)	0.476
**Pidotimod start**			
WBC count at admission, ×10^6^/µL	6820 (5950–9890)	6420 (5360–10,900)	0.773
Neutrophil count, ×10^6^/µL	5480 (4030–8740)	5310 (4220–9010)	0.865
Lymphocyte count, ×10^6^/µL	1100 (590–1370)	990 (730–1540)	0.538
NLR	6.35 (2.3–9.3)	5.4 (4.8–7.1)	0.081
NLR ≥ 6.5	8 (50)	8 (50)	1.000
**7 days post Pidotimod start**			
WBC count at admission, ×10^6^/µL	9090 (8000–10,982)	7330 (5420–12,032)	1.000
Neutrophil count, ×10^6^/µL	6920 (5280–7480)	6180 (4020–11,810)	0.076
Lymphocyte count, ×10^6^/µL	2055 (1360–3255)	1000 (750–1510)	0.003
NLR	2.9 (1.7–4.6)	5.5 (3.4–7.1)	0.037
NLR ≥ 6.5	1 (6)	6 (37.5)	0.033

Data are reported as frequencies (prevalence) and medians (inter-quartile ranges) as appropriate. NLR = neutrophil to lymphocyte ratio; WBC: white blood cell count.

**Table 3:** Statistically significant differences (*p* < 0.05) in virus-specific cytokine and chemokine production shown in Figure 2 (panel A) and in gene expression analyses shown in Figure 3 (panel B).

**A**	**Unstimulated**			**SARS-CoV-2**			**FLU**		
	**T0 vs. T1**	**T0 vs. T2**	**T1 vs. T2**	**T0 vs. T1**	**T0 vs. T2**	**T1 vs. T2**	**T0 vs. T1**	**T0 vs. T2**	**T1 vs. T2**
IL-1RA	ns	ns	ns	ns	ns	ns	0.0026671	0.050791	ns
IL-4	ns	ns	ns	ns	ns	ns	ns	ns	ns
IL-5	ns	ns	ns	0.0107572	ns	ns	ns	ns	ns
IL-6	ns	ns	ns	ns	ns	ns	0.0520084	ns	ns
IL-8	ns	ns	ns	ns	ns	ns	0.0304641	0.0212915	ns
IL-9	0.0468253	ns	ns	ns	ns	ns	ns	ns	ns
FGF-basic	ns	ns	ns	ns	ns	ns	0.0410918	ns	ns
G-CSF	ns	ns	0.025881	0.0533785	ns	ns	ns	ns	ns
GM-CSF	ns	ns	ns	ns	ns	ns	0.0520894	ns	ns
IFN-γ	ns	ns	ns	ns	ns	ns	0.0057612	0.0265163	ns
MCP-1	ns	ns	ns	ns	ns	ns	0.0025601	0.0519345	ns
MIP-1α	ns	ns	0.0419556	0.0369439	ns	ns	ns	ns	ns
MIP-1β	ns	ns	ns	ns	0.0474791	ns	ns	ns	ns
TNF-α	ns	ns	ns	0.0110859	ns	ns	ns	ns	ns
VEGF	ns	ns	ns	ns	ns	0.0524005	ns	ns	ns
**B**	**Unstimulated**			**SARS-CoV-2**			**FLU**		
	**T0 vs. T1**	**T0 vs. T2**	**T1 vs. T2**	**T0 vs. T1**	**T0 vs. T2**	**T1 vs. T2**	**T0 vs. T1**	**T0 vs. T2**	**T1 vs. T2**
TLR1	ns	ns	ns	ns	ns	ns	ns	0.0181312	0.0520254
TLR8	ns	ns	ns	ns	ns	ns	ns	0.0373754	0.0170549
HLAA	ns	ns	ns	0.0366873	ns	ns	ns	ns	ns
CCL3	ns	0.0446018	ns	0.0513275	0.0417868	ns	ns	ns	ns
CXCL10	ns	ns	ns	ns	ns	ns	0.0127521	ns	ns
IL10	ns	ns	ns	ns	0.0276853	0.0035351	ns	ns	ns
CD14	ns	0.0089948	0.0274998	ns	0.0039326	0.0350016	0.0290724	0.0017886	0.0113182
SLPI	ns	0.0492902	ns	ns	ns	ns	ns	0.0433022	ns
CAMP	ns	ns	ns	ns	ns	ns	ns	ns	0.0535916
BPI	ns	ns	0.0540071	ns	ns	ns	ns	ns	ns
IFITM1	ns	ns	ns	ns	ns	0.0407747	ns	ns	ns
HAVCR2	ns	ns	ns	ns	ns	0.0474798	ns	ns	ns
BIRK3	ns	ns	ns	ns	ns	0.0266934	ns	ns	ns
NFKB1	ns	0.0248691	ns	ns	ns	ns	ns	0.0058548	0.0509105
SIGLEC1	ns	ns	ns	ns	ns	ns	0.013669	ns	0.0138821
MPO	ns	ns	ns	ns	ns	0.0253309	ns	ns	ns

## Data Availability

Individual patient data will be available, upon individual and specific request, to researchers whose proposed use of the data has been approved. Data will be made available request to: daria.trabattoni@unimi.it and pierachille.santus@unimi.it. Data will be provided with investigator support, after approval and after signing a data access agreement. The use of individual patient data outside personal consultation will not be permitted.
